# Bifunctional Anti-Huntingtin Proteasome-Directed Intrabodies Mediate Efficient Degradation of Mutant Huntingtin Exon 1 Protein Fragments

**DOI:** 10.1371/journal.pone.0029199

**Published:** 2011-12-22

**Authors:** David C. Butler, Anne Messer

**Affiliations:** 1 Wadsworth Center, New York State Department of Health, Albany, New York, United States of America; 2 Department of Biomedical Sciences, University at Albany, Albany, New York, United States of America; Hertie Institute for Clinical Brain Research and German Center for Neurodegenerative Diseases, Germany

## Abstract

Huntington's disease (HD) is a fatal autosomal dominant neurodegenerative disorder caused by a trinucleotide (CAG)_n_ repeat expansion in the coding sequence of the huntingtin gene, and an expanded polyglutamine (>37Q) tract in the protein. This results in misfolding and accumulation of huntingtin protein (htt), formation of neuronal intranuclear and cytoplasmic inclusions, and neuronal dysfunction/degeneration. Single-chain Fv antibodies (scFvs), expressed as intrabodies that bind htt and prevent aggregation, show promise as immunotherapeutics for HD. Intrastriatal delivery of anti-N-terminal htt scFv-C4 using an adeno-associated virus vector (AAV2/1) significantly reduces the size and number of aggregates in HDR6/1 transgenic mice; however, this protective effect diminishes with age and time after injection. We therefore explored enhancing intrabody efficacy via fusions to heterologous functional domains. Proteins containing a PEST motif are often targeted for proteasomal degradation and generally have a short half life. In ST14A cells, fusion of the C-terminal PEST region of mouse ornithine decarboxylase (mODC) to scFv-C4 reduces htt exon 1 protein fragments with 72 glutamine repeats (httex1-72Q) by ∼80–90% when compared to scFv-C4 alone. Proteasomal targeting was verified by either scrambling the mODC-PEST motif, or via proteasomal inhibition with epoxomicin. For these constructs, the proteasomal degradation of the scFv intrabody proteins themselves was reduced<25% by the addition of the mODC-PEST motif, with or without antigens. The remaining intrabody levels were amply sufficient to target N-terminal httex1-72Q protein fragment turnover. Critically, scFv-C4-PEST prevents aggregation and toxicity of httex1-72Q fragments at significantly lower doses than scFv-C4. Fusion of the mODC-PEST motif to intrabodies is a valuable general approach to specifically target toxic antigens to the proteasome for degradation.

## Introduction

Huntington's disease (HD) is the most prevalent of nine known human neurodegenerative disorders linked to the expansion of polyglutamine (polyQ) tracts in specific disease-associated proteins [1]. The cellular localization of wild-type Huntingtin (htt) is predominantly cytosolic and diffuse; however, N-terminal fragments of mutant htt (mhtt) have been reported to form both intranuclear and cytoplasmic inclusions in HD [2], [3], [4]. N-terminal mhtt fragments can fold into several conformations resulting in different solubilities and pathological consequences [5], [6]. Although the precise conformations of the toxic species are still a matter of debate, it is clear that various misfolded N-terminal cleavage products are a major early step in HD pathogenesis [7], [8]. Because HD is a progressive genetic disorder with death occurring 10–20 years after diagnosis, early intervention therapies may significantly improve patient quality of life by slowing and/or reversing the course of the disease.

Intrabody-based therapies show significant potential for addressing the critical need to reduce the misfolded protein burden in HD [9]. These recombinant single-chain and single-domain variable fragments of full-length antibodies exhibit high specificity and affinity for targets, can be selected, engineered, and delivered as genes [10], [11], [12], [13]. The N-terminal 17 amino acids of htt form a highly conserved amphipathic alpha helix immediately preceding the polyQ tract, and have been shown to be involved in membrane binding, subcellular localization, aggregation, and toxicity [14], [15], [16], [17]. A naïve human spleen scFv phage-display library screened against the N-terminal 17 amino acids of htt generated the scFv-C4 intrabody, which successfully counteracts *in situ* length-dependent htt aggregation, in both cell culture [18], [19], [20], [21] and *Drosophila* models of HD [22]. scFv-C4 preferentially binds to soluble mhtt N-terminal fragments. It is only weakly active against endogenous full-length mhtt and wild type htt, possibly due to epitope inaccessibility [20]. Intrastriatal delivery of scFv-C4, using the adeno-associated virus vector (AAV2/1), resulted in a significant reduction in the size and number of mhtt aggregates in B6.Cg-HDR6/1 transgenic mice. However, the neuroprotective effect weakened both with severity of disease at time of injection, and with age beyond 6 months, although it does not disappear entirely [23]. Additional optimization of scFv-C4 is required for this intrabody to be of future use in clinical applications.

In this study, we developed a bifunctional intrabody that prevented N-terminal htt exon 1 (httex1) protein fragments from aggregating while directing them to the proteasome for degradation. Proteins that contain enriched regions of amino acids Proline (P), Glutamic Acid (E) or Aspartic Acid (D), Serine (S), and Threonine (T), otherwise known as PEST regions, are targeted for proteasomal degradation and generally have a short half-life. Mouse Ornithine Decarboxylase (mODC), a cytosolic enzyme involved in the biosynthesis of polyamines, is rapidly degraded in mammalian cells [24]. Deletion of the C-terminal PEST motif from mODC stabilizes mODC independent of protein synthesis, with no detrimental effects on enzyme activity [24]. Transfer of the mODC-PEST motif (amino-acids 422–461) to the C-terminus of stable proteins such as green fluorescent protein [25] and Luciferase [26] significantly reduced their intracellular half-life. Although there is one report in the literature that a PEST-fused intrabody against β-galactosidase was unsuccessful in depleting its target, intrabodies and targets can vary greatly in their intracellular properties, and our system differs significantly from the one reported [27]. We have previously shown that fusion of a nuclear localization sequence (NLS) derived from SV40 Large T antigen to scFv-C4 was able to bind, transport, and sequester soluble httex1 protein fragments with 72 glutamine repeats fused to enhanced green florescent protein (httex1-72Q-GFP) into the nuclei [18], [19]. Based on the ability of the scFv-C4-NLS fusion construct to redirect httex1-72Q-GFP protein to different subcellular compartments, we hypothesized that fusion of the proteolytic PEST signal sequence of mODC to scFv-C4 could affect the steady-state level of httex1-72Q-GFP by redirecting the antigen-antibody complex to the proteasome. Our current studies provide a strong proof of concept that PEST fusion can greatly enhance the functionality of our intrabody, with implications for multiple additional diseases.

## Results

### Anti-htt scFv-C4-PEST intrabody enhances degradation of httex1-72Q fragments

Our approach sought to harness the proteasomal machinery for the targeted degradation of httex1 protein fragments. To develop a bifunctional intrabody that redirected httex1 protein fragments to the proteasome for degradation, while preventing aggregation of mhtt, we fused the mODC-PEST motif (amino-acids 422–461) to the C-terminus of scFv-C4 intrabody to generate scFv-C4-PEST. Because the targeted epitope of scFv-C4 is the N-terminal 17 amino acids of htt, scFv-C4 and scFv-C4-PEST are likely to bind a variety of N-terminal mhtt fragments produced from the cleavage of full-length mhtt during HD pathology. We co-transfected intrabody constructs (empty vector, scFv-C4, and scFv-C4-PEST) with GFP-labeled huntingtin fragments of either non-pathological (httex1-25Q-GFP) or pathological (httex1-72Q-GFP) repeat lengths to determine if scFv-C4-PEST could redirect N-terminal htt fragments to the proteasome in ST14A striatal progenitor cells [28].

**Figure 1:**
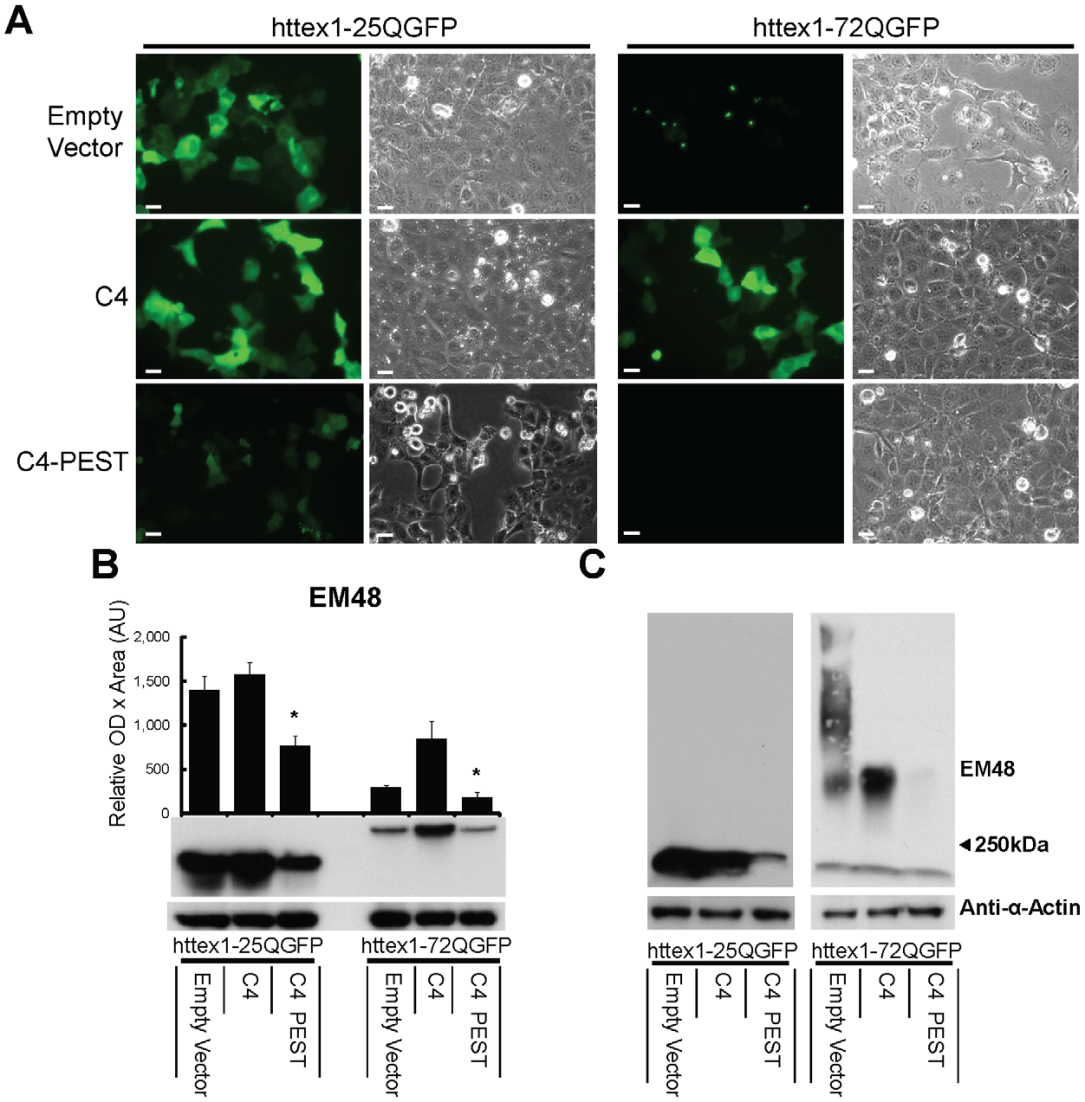
scFv-C4-PEST enhances degradation of GFP-tagged httex1 soluble and insoluble protein fragments. ST14A cells were co-transfected with GFP-tagged httex1-25Q (httex1-25Q-GFP) or (httex1-72Q-GFP), plus either empty vector, scFv-C4, or scFv-C4-PEST plasmids. **A**. Representative live cell imaging depicting reduction of httex1-72Q-GFP and httex1-25Q-GFP fluorescence in the scFv-C4-PEST co-transfected groups. Phase contrast confirms uniform cell integrity. 48 h; bar = 20 µm. **B**. SDS-PAGE Western blot and densitometry probed for htt (EM48), quantified vs. actin. Monomeric soluble mhttex1 protein fragments were quantitatively reduced in scFv-C4-PEST vs. scFv-C4 co-transfected cells. (Mean ± SEM; *p<0.05 comparing httex1-25Q-GFP co-transfections; *p<0.05 comparing httex1-72Q-GFP co-transfections) Note that **B** shows only soluble httex1-72Q fragment levels, which are low unless intrabody is present. **C**. Agarose Gel Electrophoresis for Resolving Aggregates (AGERA) shows the decrease of detergent-insoluble material in httex1-72Q-GFP to scFv-C4-PEST co-transfected cells compared to other groups.

Live cell imaging of transfected cells (Fig. 1A) revealed that without intrabody, httex1-25Q-GFP remained diffuse throughout the cell compared to httex1-72Q-GFP, which formed punctate aggregates. In cells co-transfected with scFv-C4 and httex1-72Q-GFP, GFP-labeled mhtt was diffusely localized throughout the cell, as previously reported [20], [21], [29], [30]. However, co-transfection of scFv-C4-PEST resulted in a dramatic reduction of observable httex1-25-QGFP and httex1-72Q-GFP fluorescence (Fig. 1A). Residual httex1-72Q-GFP seen after 4-fold overexposure of scFv-C4-PEST showed a phenotype consistent with native scFv-C4 (Data not shown). In agreement with live cell imaging, scFv-C4-PEST vs. scFv-C4 showed a 51% reduction of monomeric htt in httex1-25Q-GFP and a 78% reduction of httex1-72Q-GFP co-transfected cells by Western blot (Fig. 1B). To assess the effectiveness of our anti-htt intrabody based therapy on the biochemical formation of mhtt aggregates, we used agarose gel electrophoresis for resolving aggregates (AGERA). Unlike sodium dodecyl sulfate polyacrylamide gel electrophoresis (SDS-PAGE), where aggregated mhtt is trapped in the stacking gel, AGERA allows aggregated mhtt to be separated based on size, with larger aggregates remaining near the top of the gel [31]. In cells co-transfected with empty vector and httex1-72Q-GFP, there was a large smear of aggregated mhtt compared to cells co-transfected with scFv-C4; however, in cells co-transfected with scFv-C4-PEST aggregated htt was substantially reduced with respect to empty vector and scFv-C4 transfected cells (Fig. 1C, right panel). scFv-C4-PEST appeared to remove httex1-25Q-GFP as well, but presumably at the level of monomer (Fig. 1C, left panel).

### scFv-C4-PEST inhibits polyQ-mediated toxicity of mutant httex1-72Q-GFP protein fragments

**Figure 2:**
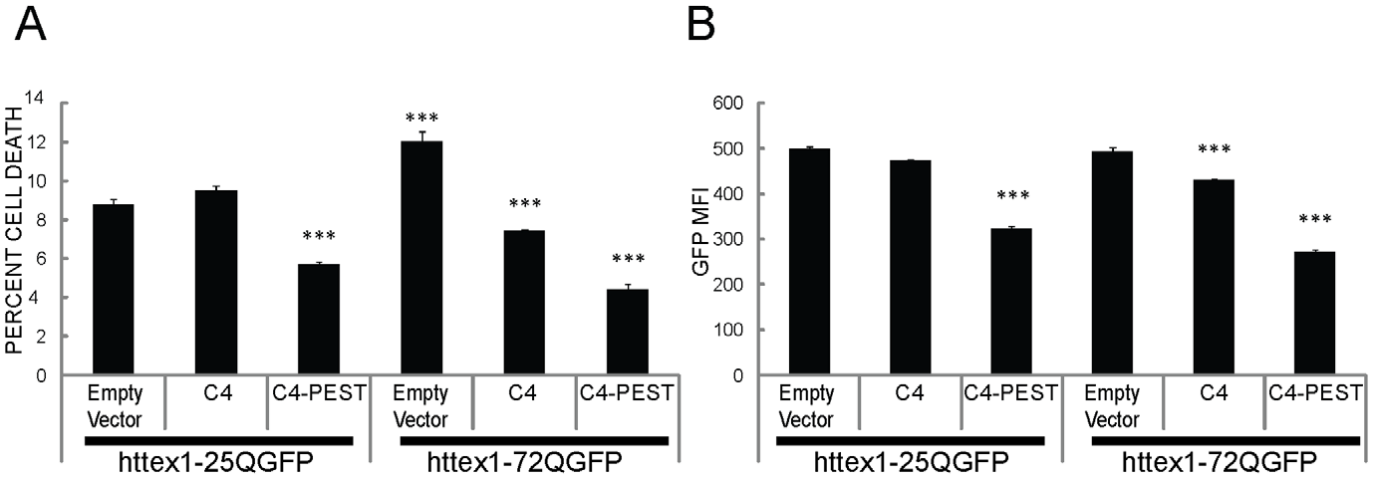
scFv-C4-PEST further enhances viability in ST14A co-transfection experiments compared to scFv-C4. Viability and GFP geometric mean fluorescent intensity (MFI) were assayed by flow cytometric analysis 48 h after transfection. For scFv-C4-PEST vs.scFv-C4: **A**. The percentage of propidium iodide (PI) positive nonviable cells is reduced. **B**. Total httex1-25Q-GFP and httex1-72Q-GFP reduction was confirmed. (means ± SEM; *** p<0.001 within groups.)

To further examine functionality, viability was assayed by flow cytometric analysis with propidium iodide (PI). As seen in previous studies, we observed an increase of PI-positive non-viable cells when empty vector was co-transfected with httex1-72Q-GFP compared to cells co-transfected with httex1-25Q-GFP (Fig. 2A) [18]. Both scFv-C4 and scFv-C4-PEST inhibited polyQ-mediated toxicity of mutant httex1-72Q-GFP protein fragments (Fig. 2A). In agreement with findings reported in Figure 1, scFv-C4-PEST significantly reduced the geometric mean fluorescence intensity (MFI) of GFP labeled httex1-25Q and httex1-72Q cells by 1.5 and 1.8-fold, respectively (Fig. 2B).

### PEST scramble and specific inhibition of the proteasome eliminates the enhanced degradation of N-terminal httex1-72Q-GFP protein fragments

The solubility of human scFv intrabodies can be improved by an overall negative charge at cytoplasmic pH and increased hydrophilicity (as demonstrated by decreased grand average of hydropathicity, GRAVY score) [32]. Our previous data show that addition of a highly charged acidic 3×FLAG tag to aggregation-prone positively charged intrabodies resulted in increased intracellular solubility [32]. Because a PEST sequence is defined by hydrophilic stretches of amino acids greater than or equal to 12 residues in length [33], addition of a PEST motif to scFv intrabodies may enhance their intracellular solubility. Sequence analyses of scFv-C4 and estimation of net charge at pH 7.4 GRAVY hydrophilicity score predicted that fusion of hydrophilic PEST motif to scFv-C4 could increase solubility (Table 1). To determine if the enhanced clearance of httex1-72Q-GFP protein fragments was simply due to enhanced solubility of scFv-C4, we scrambled the PEST motif (scFv-C4-PEST-SCR), while maintaining the physico-chemical properties of scFv-C4. Because positively charged residues are not contained within PEST regions [33], we rearranged the PEST motif placing positively charged amino acids within the PEST motif. The subsequent PEST-FIND scores for scFv-C4-PEST and scFv-C4-PEST-Scramble were +5.16 and −2.41 respectively (Table 1).

**Table 1:** Physico-chemical properties of scFv-C4 intrabodies.

	Isoelectric point	Net Charge at pH 7.4	Gravy Score	PEST Find Score
scFv-C4	6.62	−0.5	−0.282	------
scFv-C4-PEST	5.24	−5.5	−0.328	5.16
scFv-C4-PEST-SCR	5.24	−5.5	−0.328	−2.41

Physico-chemical determinants of soluble intrabody expression. Solubility is improved by an overall negative charge at pH 7.4 and reduced GRAVY score. The GRAVY score is the average hydropathy score for all the amino acids in the protein, with a more negative score indicating increased hydrophilicity. The PEST-FIND program http://emboss.bioinformatics.nl/cgi-bin/emboss/pestfind objectively produces a score ranging from about −50 to +50. A score above +5 denotes a PEST region [33].

**Figure 3:**
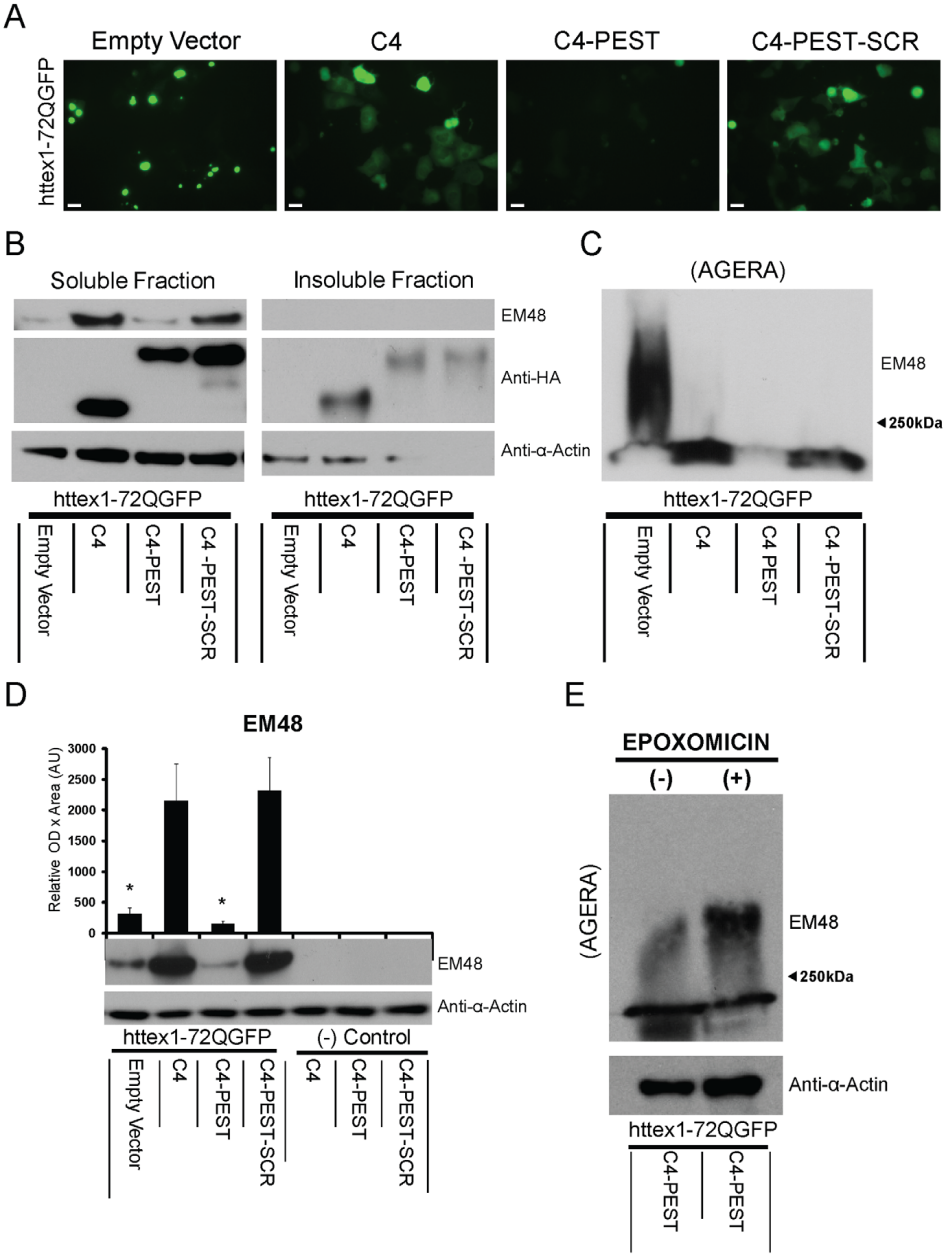
Scrambling the PEST sequence and specific inhibition of the proteasome eliminates the enhanced degradation of httex1-72Q-GFP. **A**. Representative live cell images of dual transfections. Diffuse httex1-72Q-GFP fluorescence is similar between scFv-C4-PEST-SCR and scFv-C4 cells, and much higher than scFv-C4-PEST. **B**. Biochemical fractionation of HA-tagged fluorobodies from ST14A cells. Detergent-soluble and -insoluble cell lysates were prepared as described in Materials and Methods. Empty vector and scFv-C4-PEST transfected cells express minimal levels of monomeric htt, while scFv-C4-PEST-SCR and scFv-C4 transfected cells have similar levels of monmeric htt **C**. AGERA. There is an increase of detergent-insoluble material in scFv-C4-PEST-SCR transfected cells compared to scFv-C4-PEST. **D**. Western blotting of total protein. Monomeric mhtt levels do not differ between scFv-C4 and scFv-C4-PEST-SCR co-transfected cells. C4-PEST reduces soluble mhtt by ∼90%, and empty vector control mhtt is insoluble **E**. Proteasome inhibition reduced clearance of insoluble httex1-72Q by scFv-C4-PEST. ST14A cells were co-transfected with httex1-72Q-GFP and scFv-C4-PEST. 36 h after transfection, cells were treated with 10 µM epoxomicin (+), a potent and selective proteasome inhibitor [34]; or DMSO vehicle control. Insoluble mhtt was assessed at 48 h using AGERA. Proteasome inhibition resulted in reduced clearance of htt exon1 protein fragments by scFv-C4-PEST.

To determine if the enhanced clearance of httex1-72Q-GFP protein fragments was due to enhanced scFv-C4-PEST intrabody protein solubility, we examined protein levels of the different intrabody constructs. There appears to be no qualitative difference in the solubility of HA tagged scFv-C4, scFv-C4-PEST, or scFv-C4-PEST-SCR intrabodies, based on biochemical fractionation into detergent-soluble and –insoluble cell lysates (Fig. 3B). These data suggest that enhanced solubility of scFv-C4-PEST is not a major factor in PEST-mediated enhanced target clearance.

Live cell imaging of scFv-C4-PEST-SCR produced similar results to scFv-C4 for inhibiting aggregation of httex1-72Q-GFP (Fig. 3A). In agreement with findings reported in Figure 1, monomeric mhtt was significantly reduced in empty vector control and scFv-C4-PEST detergent-soluble fractions (Fig. 3B). As expected, the levels of monomeric mhtt were similar between scFv-C4 and scFv-C4-PEST-SCR (Fig. 3B). The absence of monomeric mhtt from the insoluble fractions is due to aggregation of httex1-72Q-GFP, which is retained in the stacking gel. To address this issue, total cell lysates were analyzed by AGERA. Empty vector and httex1-72GFP co-transfected cells contain a substantial amount of detergent insoluble mhtt compared to all other groups (Fig. 3C, Left lane). As expected the levels of detergent insoluble mhtt are similar between scFv-C4-PEST-SCR and scFv-C4 compared to scFv-C4-PEST co-transfected cell lysates (Fig. 3C). Immunoblot quantification of this series showed that there was a 90% reduction of soluble mhtt in scFv-C4-PEST cells compared to scFv-C4 and scFv-C4-PEST-SCR (Fig. 3D).

To further confirm that scFv-C4-PEST is acting in a proteasome-dependent manner, we treated cells with epoxomicin, a potent and selective proteasome inhibitor [34]. ST14A cells were co-transfected with httex1-72Q-GFP and scFv-C4-PEST. 36 h after transfection, cells were treated with 10 µM epoxomicin or vehicle control DMSO, and then harvested at 48 h. As expected, 12-h proteasome inhibition with epoxomicin resulted in reduced clearance of insoluble mhtt in scFv-C4-PEST and httex1-72Q co-transfected cells (Fig. 3E).

### PEST fusion reduces steady-state scFv-C4 intrabody levels modestly, and independent of antigen

**Figure 4:**
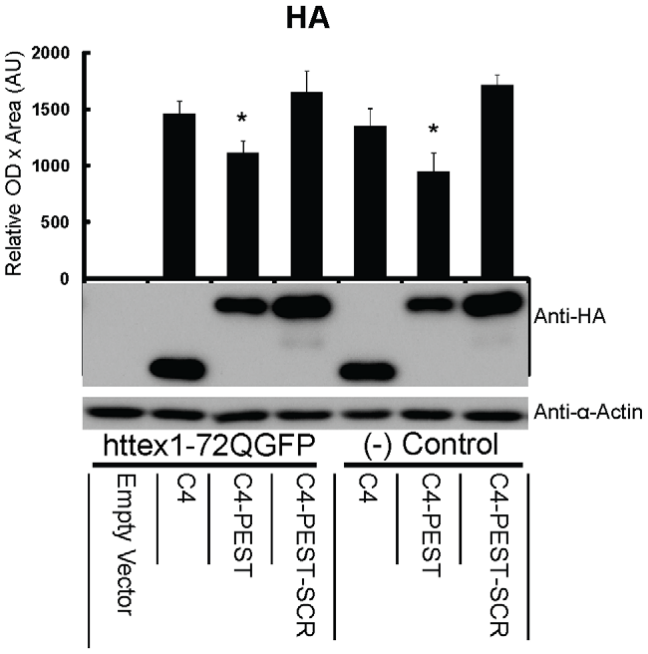
PEST fusion reduces steady-state scFv-C4 intrabody levels modestly, and independent of antigen. Using dual transfections as above, scFv-HA levels were compared among scFv-C4, scFv-C4-PEST, and scFv-C4-PEST-SCR, in the presence and absence of httex1-72Q-GFP. Levels of scFv-C4-PEST were reduced by ∼25% when compared with scFv-C4 or the scrambled (inactive) PEST. The presence or absence of antigen had no significant effect on intrabody-PEST levels. (n = 4; mean ± SEM; *p<0.05. for C4 vs. C4-PEST/C4-PEST-SCR.)

**Figure 5:**
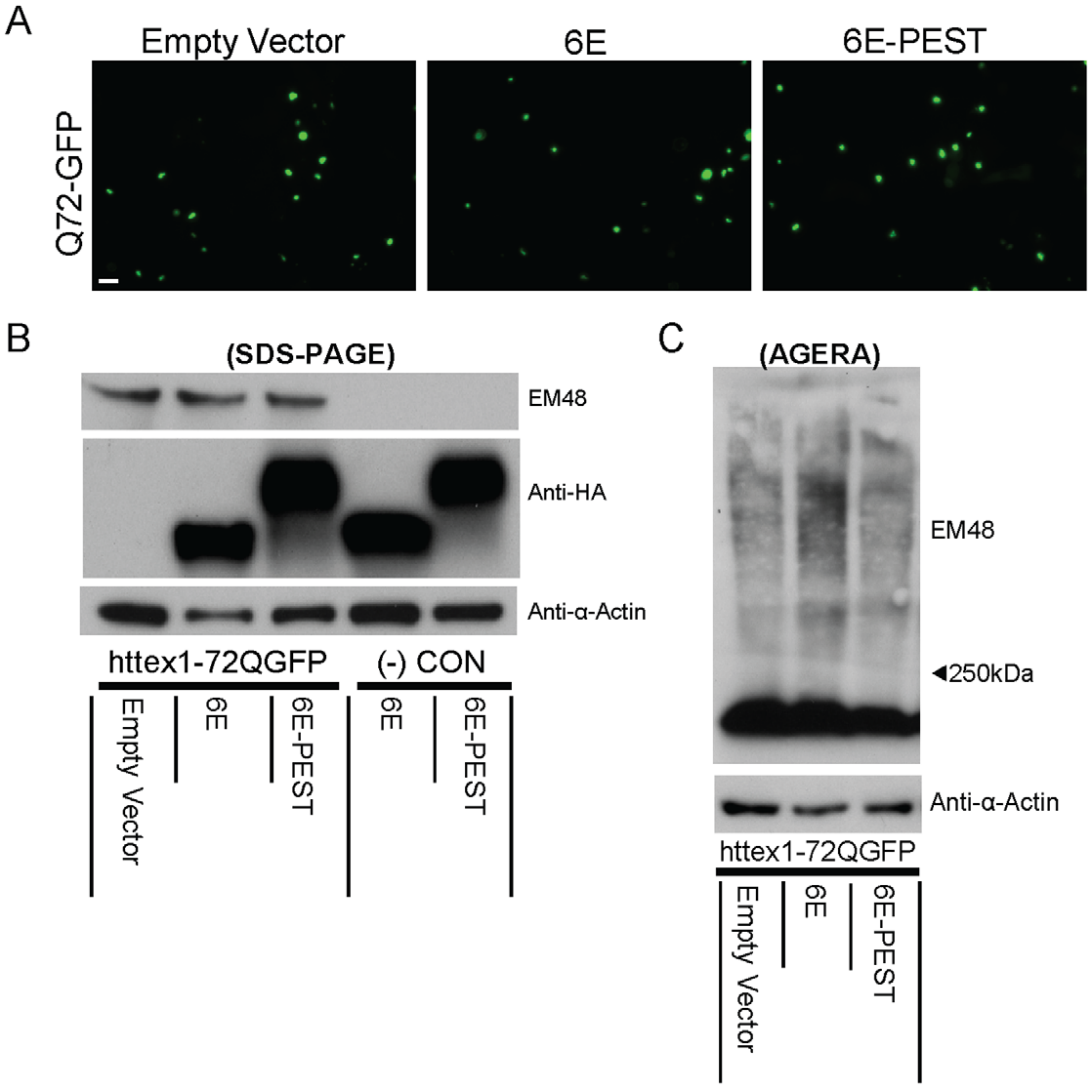
Fusion of PEST motif to anti htt fibril-specific scFv-6E intrabody does not degrade htt fragments in ST14A cells. ST14A cells co-transfected with httex1-72Q-GFP and either empty vector, scFv-6E, or scFv-6E-PEST plasmids. Cells imaged and processed at 48 h. **A**. Live cell imaging. scFv-6E-PEST does not appear to alter the aggregation of htt compared to empty vector or scFv-6E. **B**. Western blotting. Monomeric htt is similar between groups. HA-tagged intrabody levels are similar between groups. **C**. Detergent-insoluble material resolved by AGERA is similar between empty vector and 6E-PEST groups.

Sibler et al. reported that fusion of the mODC-PEST motif to the anti- β-galactosidase scFv-13R4 intrabody resulted in a destabilization of the intrabody-PEST construct in the absence of intracellular target [27]. The experiments above also allowed us to determine if fusion of the mODC-PEST motif to the C-terminus altered the steady state levels of anti-htt scFv-C4. In Fig. 4, we quantitatively compared the expression levels of scFv-C4 and scFv-C4-PEST with and without intracellular target httex1-72Q-GFP, using a hemagglutinin (HA) tag to identify the intrabodies. In contrast to scFv-13R4-PEST intrabody, our steady-state protein levels of scFv-C4-PEST were reduced by only 25% independent of antigen presence or absence. Likewise, the levels of a second intrabody, anti-fibrillar scFv-6E and scFv-6E-PEST are similar with and without intracellular target httex1-72Q-GFP (Fig. 5B). Interestingly, scFv-13R4, scFv-C4 and scFv-6E were derived from different intrabody libraries. These data suggest that not all classes of intrabodies are significantly destabilized by the addition of the mODC-PEST motif.

### PEST mediated degragation of mhtt is specific to soluble monomeric, and not fibrillar, species

Insoluble polyQ protein aggregates are generally resistant to proteolytic degradation [35]. We fused the mODC-PEST motif to a fibril-specific scFv-6E intrabody [36], which does not bind to the soluble monomeric mhtt fragment [18], to investigate whether an aggregated/fibrillar htt-PEST complex is also resistant to proteolytic degradation. Previously, we reported that under slightly different conditions, scFv-6E modestly enhanced the aggregation propensity of httex1-72Q-GFP compared to httex1-25Q-GFP in ST14A cells [18]. As opposed to the soluble monomer binding scFv-C4 intrabody studies above, live cell imaging of httex1-72Q-GFP is qualitatively indistinguishable when PEST is fused to the anti-fibrillar scFv-6E intrabody (Fig. 5A). Western blotting confirmed that the levels of monomeric mhtt were comparable among empty vector, scFv-6E, and scFv-6E-PEST (Fig. 5B). Detergent-insoluble migration patterns of httex1-72Q-GFP resolved by AGERA were also similar between empty vector, scFv-6E, and 6E-PEST through the gel (Fig. 5C). These data are consistent with previous studies that suggest aggregated htt is inefficiently degraded by the proteasome [37].

### scFv-C4-PEST alters target at a lower dose than scFv-C4

**Figure 6:**
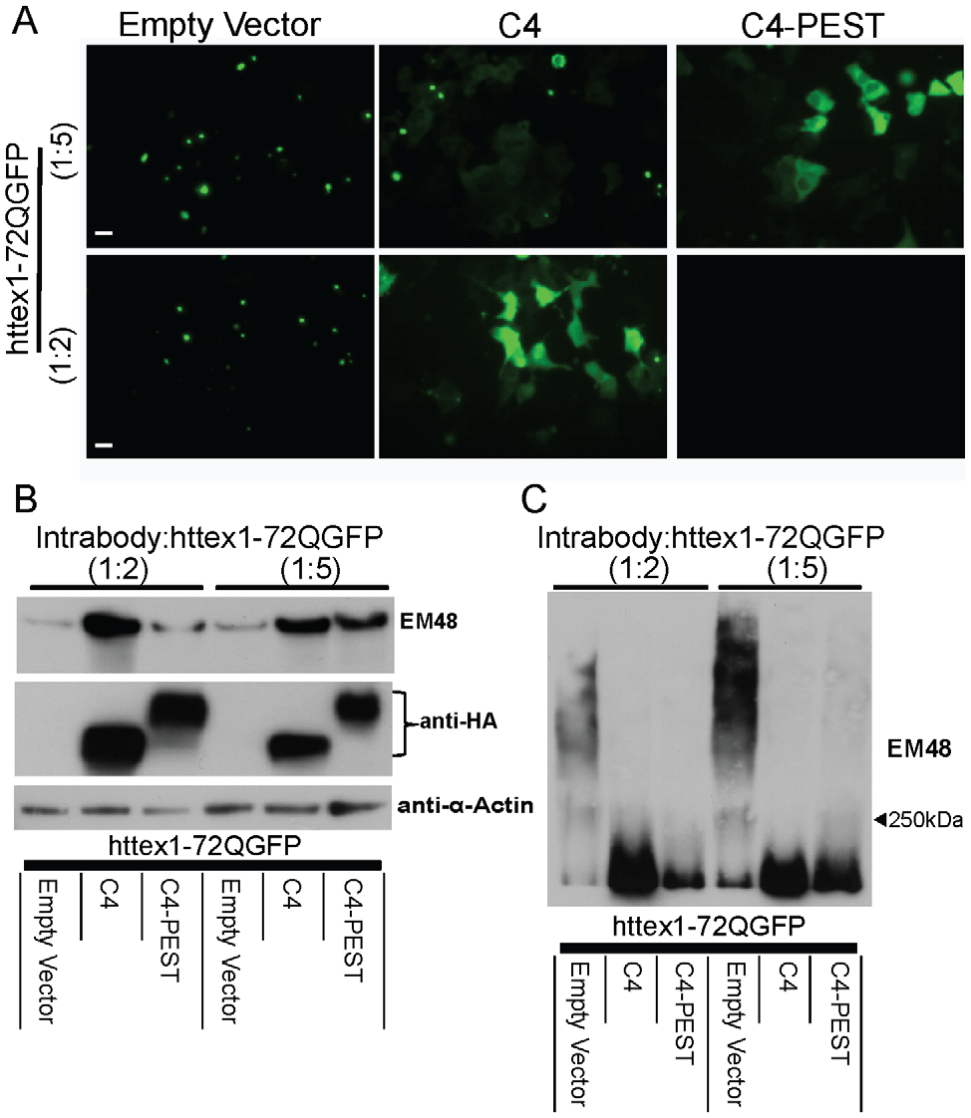
Targeted degradation of htt occurs in a dose-dependent manner. ST14A cells co-transfected with 1∶2 or 1∶5 transfection ratio of intrabody-PEST to httex1-72Q-GFP plasmids. **A**. Live cell imaging. Httex1-72Q is diffuse at 1∶2, and being begins to form aggregates at a 1∶5 transfection ratio of intrabody to httex1-72Q-GFP in scFv-C4 transfected cells. Fusion scFv-C4-PEST clears mhtt at 1∶2, and keeps mhtt diffuse at 1∶5. **B, C**. Western blots of EM48 immunoreactivity measuring the soluble (**B**; SDS-PAGE) and insoluble (**C**; AGERA) material to confirm live cell imaging.

A therapeutic range is especially advantageous for gene therapy. We therefore co-transfected ST14A cells with a 1∶2 or a 1∶5 ratio of intrabody to httex1-72Q-GFP. In contrast to previous experiments (see Fig. 1A), where intrabody and httex1-72Q-GFP were co-transfected at a 1∶1 ratio, aggregation of httex1-72Q-GFP was present in scFv-C4 transfected cells at a 1∶2 ratio and was noticeably elevated at a 1∶5 ratio (Fig. 6A). However, scFv-C4-PEST transfected cells still enhanced the clearance of httex1-72Q-GFP at a 1∶2 ratio of intrabody to httex1-72Q-GFP. At a 1∶5 ratio of scFv-C4-PEST to httex1-72Q-GFP, scFv-C4-PEST maintained httex1-72Q-GFP in a mostly non-aggregated soluble state (Fig. 6A). Western blots of the dose response experiments confirmed the live cell observation that scFv-C4-PEST can counteract aggregation very effectively at 1∶2, and still shows effects at 1∶5 (Fig. 6B). Compared to scFv-C4, the levels of insoluble httex1-72Q-GFP were reduced at 1∶2 and 1∶5 ratio of scFv-C4-PEST to httex1-72Q-GFP (Fig. 6C).

## Discussion

In HD, the initial pathogenic trigger is the misfolding of mhtt, which eventually leads to intracellular accumulation of a toxic N-terminal fragment of the mhtt protein with excess polyQ. Prevention of mhtt misfolding, or reducing its availability for abnormal intracellular interactions, should therefore offer significant protection. We and others have developed intrabodies that bind to the regions adjacent to the polyQ repeat [38], [39], [40], altered the protein context, and reduced the aggregation and toxicity of mhtt. One very promising candidate, the scFv intrabody scFv-C4, targets the N-terminal 17 amino acids of the htt protein, a domain of htt protein that is increasingly being recognized as pivotal in mediating physiological and pathological pathways in HD [41]. The human scFv-C4 is very effective at counteracting the misfolding and toxicity of mutant httex1 in transient transfection assays of multiple cell lines [10] and stressed organotypic brain slice cultures [21]. In an HD *Drosophila* model, the intrabody shows strong protective effects initially, and increases lifespan by 30%, although the mutant flies still die prematurely [42]. AAV2/1 delivery of this intrabody to the striatum of inbred B6.HDR6/1 mice also demonstrates phenotypic correction at the cellular level; however, scFv-C4 alone has been insufficient to produce amelioration of the full disease phenotype over a period of several months [23]. This could be due to targeting of a suboptimal epitope, gradual dilution of efficacy due to build-up of mhtt that escapes intrabody binding, and/or insufficient delivery of an otherwise effective reagent. Work here and elsewhere suggests that all three of these parameters impact long-term efficacy *in vivo*. Here, we addressed the first two factors by engineering fusions of scFv-C4 to heterologous proteolytic domains that can lead to rapid and irreversible turnover of the antigen-antibody complex. Inducing rapid turnover of the bound antigen reduces dependence on the precise functionality of the bound epitope, although the epitope must still be available. We show that fusion of scFv-C4 to a proteasomal PEST signal domain results in greatly enhanced degradation of soluble mutant httex1 protein fragments in a transiently-transfected striatal cell line. This in turn reduces both the aggregate burden and the toxicity of the mutant protein.

The goal of this study was to determine whether a bifunctional intrabody could manipulate the stability of its intracellular target. Because heterologous transfer of the mODC-PEST motif to the C-terminus of stable proteins significantly reduced their intracellular half-life [25], [26], fusion of a PEST motif to scFv-C4 might destabilize the intrabody protein itself, thus reducing the amount of intrabody available to bind to its target. For this reason, we chose a specific mODC-PEST sequence that was known to induce moderate, rather than very strong, turnover, based on destabilized GFP-PEST studies [25]. It was also important to consider the functional state of the proteasome in affected cells. The ubiquitin-proteasome system (UPS) has been implicated in the failure to degrade mhtt, leading to aggregate formation [43]. Proteasome impairment is thought to occur through sequestering of 20S proteasome subunits [37], failure of aggregated mhtt to enter the 20S proteasome core [44], or clogging of the proteasome with polyglutamine fragments [45]. Fortunately, *in vivo* studies have shown that 26S proteasome is not impaired in HDR6/2 mice [44], and it is the 26S proteasome that recognizes the PEST motif of mODC and processes it for degradation [46]. Therefore, our strategy was designed to take advantage of this conserved functionality.

The difference in the ratios of scFv-C4 to scFv-C4-PEST intrabody levels vs. httex1-72Q-GFP+scFv-C4 to httex1-72Q-GFP+scFv-C4-PEST was larger than expected. Our data suggest that this particular combination of intrabody and PEST does not lead to rapid degradation of the unliganded intrabody fused to PEST. With intrabody binding, the mhtt antigen appears to be delivered to the proteasome and degraded, while sufficient intrabody-PEST protein remains available for additional substrate. Qualitative comparisons of cellular protein levels of a second intrabody, scFv-6E, which binds only to aggregated fibrillar httex1, also show intrabody levels that appear even less reduced by fusion with this PEST motif. However, in this case the fibrillar antigen-antibody complex could not be degraded by the proteasome, which is in agreement with data suggesting that once htt has aggregated it is resistant to degradation by the UPS [35]. Published results suggest that these types of highly structured aggregates are degraded via autophagy [43], [47].

For safety reasons, it would be valuable to use an intrabody that preferentially targets N-terminal mhtt fragments, because long-term reduction of full-length wild-type htt may exacerbate neurological degeneration, even in the presence of low levels of mhtt [48] We have previously shown that scFv-C4 preferentially binds to soluble mhtt N-terminal fragments, and is only weakly active against endogenous full-length mhtt and wild type htt, possibly due to epitope inaccessibility [20]. Pilot experiments using the scFv-C4-PEST construct in B6.Cg-R6/1 HD and wild-type mouse striatum have not shown excess transgene-induced toxicity up to 6 months after injection of AAV2/1-scFv-C4-PEST (data not shown). However, quantitation of the level of residual wild-type full-length htt will be necessary prior to extending these studies to clinical use.

An additional safety/efficacy issue for bifunctional intrabodies is subcellular localization. Soluble mhtt appears to be largely cytoplasmic, while aggregated mhtt is often found juxtaposed to the nucleus in ST14A cells [18]. In contrast to *in situ* studies of N-terminal mhttex1 fragment localization, aggregated mhtt is primarily localized in the nucleus of B6.Cg-R6/1 HD transgenic mice. Localization of htt is thought to be due primarily to a motif within the AA1–17 region that acts as a cytoplasmic retention signal [15], [17], [49], [50], [51]. Intrabodies that bind AA1–17 might influence this distribution by masking nuclear export or cytoplasmic retention signals, thereby increasing nuclear concentrations and toxicity. scFv-C4, which was selected against AA1–17, appears to bind to the far N-terminus, AAs 2–12 (Thumfort and Ingram, pers. Comm.). scFv-C4 does not lead to nuclear concentration of diffuse httex1-72Q-GFP, as shown here and in our previous studies. Another engineered intrabody, V_L_12.3, was selected against a peptide that included AAs1–20 [29], [30], and the epitope has recently been identified as including AAs 5–18 [52]. This binding therefore is likely to block the phosphorylation at serine 13 and serine 16, which fits well with *in situ* and *in vivo* observations that the V_L_12.3-mhttex1 fragment complex is preferentially found in the nuclear compartment, and may not be protective *in vivo* reviewed in Butler et al 2011 [9]. It would be interesting to test whether a PEST fusion of this intrabody can utilize nuclear proteasomes for mhtt degradation.

We were able to confirm that N-terminal mutant httex1 fragment degradation is dependent upon proteasomal function, since epoxomicin, a potent and selective proteasome inhibitor [34], inhibited the turnover. The intrabody-PEST mediated turnover of intracellular target is dependent upon the specific order of key amino acids within mODC-PEST motif rather than enhanced solubility. The intracellular stability of scFv-intrabodies is notoriously poor due to macromolecular crowding and an unfavorable redox potential within the cellular cytoplasm [10], [29], [53]. We have previously reported that cis-acidification of a poorly soluble scFv-intrabody with a C-terminal 3X-Flag tag can enhance intrabody solubility and function [32], thus it is possible that the addition of this highly acidic domain would improve the scFv-C4 intrabody function via enhanced folding effects. However, a scrambled version of the PEST used here maintained the charge density without enhancing the intrabody-mediated turnover of its target. Since both scFv-C4 and scFv-6E are soluble in the cellular environment, it is unlikely that enhanced solubility contributed to the clearance of htt fragments.

Intrabody-mediated degradation with a PEST motif had been previously tested with a fusion intrabody scFv-13R4 against β-galactosidase [27]. In that case, PEST fusion rendered the intrabody-PEST protein itself unstable, and binding to its target stabilized the complex. The (β-gal) target protein is extremely large, which may account for the inability of cellular proteasomes to degrade the antigen-antibody complex. The scFv-13R4 intrabody itself also belongs to a different framework family than scFv-C4 [54]. The scFv-C4 intrabody was selected from a natural non-immune human library [55], while scFv-6E used a consensus framework [56], [57]. The biophysical and intracellular properties of intrabodies are quite dependent upon both the frameworks and the complementary determining regions. This should carry over to the properties of the fusion intrabodies as well. We have previously reported that the anti-β-gal scFv-13R4 parent intrabody also differs from anti-htt scFv-C4 in solubility calculations [32]. In an attempt to circumvent the potential effect of a proteasomal targeting signal on steady-state intrabody levels, Mechionna and Cattaneo created inducible suicide intrabodies by fusion with IκBα, which must be activated by TNFα [58]. After activation, scFv-R4-IκBα reduced β-gal by 15–20%, while an anti-Tau scFv#2 reduced τ151–422 by 70–90%. However, as noted in their publication, the requirement to use a biologically active ligand is suboptimal, due to potential side-effects. It is clear from our work that there may be many classes of intrabodies that do not require exogenous activation in order to effectively degrade targets. Testing of constitutively active intrabody fusions is quite straightforward, allowing empirical determination of the most suitable fusions of intrabodies and PEST domains for specific targets. Once a sufficient number of studies have been performed on diverse constructs, it may also be possible to predict the behavior of fusions and engineer them to degrade targets without suicide. Fusion construct approaches should empower long-term intrabody-mediated correction, and may be more generally applicable to intrabody therapeutics of neurological diseases characterized by abnormal protein accumulation.

## Materials and Methods

### Expression plasmids

cDNA, previously described by Kvam et. al., [18] encoding scFv-C4 (GenBank accession number EU490426) was PCR amplified using complementary primers. The forward primer (5′-TGCTCTAGACGCCATGGCCCAGGTG CAGC-3′) introduced an XbaI restriction site (underlined) prior to the existing 5′ Kozak sequence. The reverse primers (Standard: 5′-CCC**AAGCTT**TTACTACACATTG ATCCTAGCAGAAG CACAGGCTGCAGGGTGACGGTCCATCCCGCTCTCCTGGGCACAAGACATGGGCAGCGTGCCATCATCCTGCTCCTCCACCTCCGGCGGGAAGCCATGGCTCGCGTAGTCTGGGACGTCG-3′; “Scramble”: 5′-CCC**AAGCTT**TTACTACAGCCTGTGACGATGCACTGCAGACATAGGGGCAGCAGAGTCCCCGCTCTGGATATTGGCCTCACAAGCCATGGGCGTACAGAACTCATCCTCGCCATCCTCCACCGGCTGC GGGCTGCCCGCGTAGTCTGGGACGTCG-3′) introduced a standard or scrambled PEST motif corresponding to amino acids 422–461 from mouse ornithine decarboxylase (GenBank accession number NM_013614.2) and a HindIII (BOLD) restriction site immediately following the stop codon. The resulting PCR product was ligated into pAAV-MCS (Stratagene) at the corresponding Xba1 and HindIII restriction sites using standard cloning techniques. scFv-6E (GenBank accession number FJ695518) was cloned into pAAV-MCS in a similar manner. To create fluorescently-labeled intrabodies, scFv-C4 -HA-PEST was PCR amplified using a complementary forward primer (5′-TGCTCTAGACGCCATGGCCCAGG-3′) and a reverse primer that introduced a BamHI (underlined) restriction site (5′-CGGGATCCCACATTGATCCT AGCAGAAGCAC-3′). The PCR removes the stop codon, allowing the scFv-C4 -HA-PEST construct to be ligated in-frame 5′ to a (Gly_4_Ser)_4_ flexible peptide repeat linked to a humanized enhanced green fluorescent protein (EGFP), all on a pcDNA3.1(-) plasmid backbone previously described by Kvam et al. [18]. The resulting expression plasmid cassette was the following: Kozak sequence-scFv-C4-HA-PEST-(Gly_4_Ser)_4_-EGFP-stop. Vectors for the expression of human httex1 with different polyglutamine repeat lengths labeled with either EGFP or RFP respectively, were described elsewhere (pcDNA3.1-Httex1-25Q-EGFP, pcDNA3.1-Httex1-72Q-EGFP [59], and pcDNA3.1-Httex1-25Q-RFP, pcDNA3.1-Httex1-72Q-RFP [60]. All expression plasmids were prepared using EndoFree Plasmid Maxi (Qiagen) and confirmed by DNA sequencing.

### Cell culture and transfection

Undifferentiated ST14A cells were cultured according to standard protocols [28]. ST14A cells were transiently transfected with jetPEI DNA transfection reagent (Polyplus Transfection Inc.) as previously described [18]. For all transfections, intrabody plasmids were applied at equal (1∶1) or lesser ratios (1∶2 or 1∶5) to httex1 plasmids, and cells were analyzed 48 hours post-transfection.

### Cell fractionation, AGERA, and Western blot

Transiently transfected ST14A cells were collected from 6-well culture dishes by trypsinization and washed twice with PBS. Soluble and insoluble protein fractions were isolated as previously described [18]. Briefly, whole-cell lysates were extracted at 4°C in 50 uL/well of RIPA lysis buffer (50 mM Tris pH 7.5, 150 mM NaCl, 1% NP40, 0.25% sodium deoxycholate, 0.1% SDS) supplemented with complete protease inhibitor cocktail (Roche). Detergent-insoluble material was pelleted by microcentrifugation (13,000 rpm 610 min., 4°C). Soluble protein in the supernatant was quantified using Bio-Rad DC Protein Assay kit (Bio-Rad Laboratories). Lysates were adjusted to 1 mg/mL in 2X denaturing sample buffer (125 mM Tris, 4% SDS, 20% glycerol, 10% 2-mercaptoethanol, 0.02% bromophenol blue, pH 6.8) and boiled. Insoluble pellet fractions were washed with RIPA, resuspended in 50 µL/well of 2X denaturing sample buffer, and boiled for 15 min. Equal volumes of pellet and soluble fractions corresponding to, 20 µg of total protein (10 mL) were resolved by SDS-PAGE and transferred onto PVDF membranes (PerkinElmer).

Aggregated huntingtin was quantified by agarose gel electrophoresis for resolving aggregates (AGERA) method [31]. Briefly, samples were homogenized in 10 volumes (w/v) tris-saline (10 mM Tris-HCl, pH 8.0, 150 mM NaCl, 2% SDS) and complete protease inhibitor (Roche Diagnostics) and stored at −80°C. For 1.5% agarose gels, 1.5 g agarose (Invitrogen) was dissolved in 100 mL 375 mmol/L Tris-HCl, pH 8.8 and then boiled. After melting, SDS was added to a final concentration of 0.1% (w/v). Samples were diluted 1∶1 into 2X non-denaturing sample buffer (150 mmol/L Tris-HCl pH 6.8, 33% glycerol, 1.2% SDS and bromophenol blue) and incubated for 5 min. at 95°C. Forty µg of protein was loaded per AGERA lane. After loading, gels were run in Laemmli running buffer (192 mmol/L glycine, 25 mmol/L Tris-base, 0.1% (w/v) SDS) at 100 V, 2 A until the bromophenol blue running front reached the bottom of the gel. A Transblot SD semi-dry electroblotter (BIORAD) was used to blot the gels on PDVF membranes (Millipore) at 200 mA for 1 hour. Membranes were then developed using mouse monoclonal EM48 (1∶1000, Chemicon). HA-tagged intrabodies and endogenous actin were probed with monoclonal anti-HA (1∶5,000, Covance) and anti-actin (1∶1000, Sigma) Abs, respectively, labeled with HRP-conjugated goat-anti-mouse IgG (1∶2000, Santa Cruz), and detected by ECL (PerkinElmer).

### Live-cell imaging and aggregation counts

ST14A cells transfected with GFP- and/or mRFP-tagged reporters were imaged directly in 6-well culture dishes using an Olympus IX70 inverted microscope equipped with an Olympus IXFLA Inverted Reflected Light Fluorescence Observation attachment and RGB Mirror Cube filter wheel (Olympus). Cells were observed without fixation using a 406lens, and images were captured 48 hours post-transfection with a SPOT RT Color CCD camera using SPOT Advanced software (Diagnostic Instruments). Digital images were overlayed and cropped using Adobe Photoshop.

### Flow cytometry

ST14A cells were seeded into 6-well culture dishes (2.50×10^5^ cells/well) and transfected, 24 hours later, in triplicate wells, with plasmids encoding GFP (containing either normal or expanded polyglutamine repeat tracts) and either intrabody or empty vector. Approximately 48 hours post-transfection, cell medium was harvested and adherent cells were collected by trypsinization. Harvested cells and exhausted media were combined and passed through a 70 µm cell strainer (BD Biosciences). Cells were pelleted by centrifugation (800 rpm, 5 min.) and resuspended in 1 mL of FACS buffer (16 Ca2^+^- and Mg2^+^-free PBS, 10% FBS) containing 50 mM propidium iodide (PI; Sigma). Cells were stained for 20 min. in the dark and pelleted by centrifugation (4,000 rpm, 5 min.). Cell pellets were resuspended in 0.5 mL of FACS buffer/well and transferred to Falcon 5 mL round-bottom polystyrene FACS tubes. Labeled cells were sorted by fluorescence using a BD FACSCalibur Flow Cytometer (BD Biosciences) and a minimum of 30,000 events were recorded per sample using BD CellQuest Pro software. GFP was detected with an FL1 detector, and PI with FL3. Prior to each experiment, the flow cytometer was calibrated against three control groups: (1) unstained ST14A cells to gate autofluorescence, (2) unstained ST14A cells expressing httex1-25Q-eGFP alone to identify transfected cells, and (3) stained ST14A cells pre-treated with H_2_O_2_ for maximal PI uptake. Using BD CellQuest Pro software, data were displayed in two-color dotplot formats on a log-scale, and cell death was expressed as a percentage by dividing the number of cells that stained with PI by the total number of events. Because scFv-C4-PEST decreased the MFI of GFP, gating for GFP positive cells and PI-positive cells for viability was not possible.

### Statistical analysis

All values represent the mean of at least three independent experiments, with multiple individual values per experiment. Statistical significance was determined by one-way ANOVA using Minitab or Statview statistical software. P values<0.05 were designated as statistically significant.

## References

[1] Gusella JF, MacDonald ME (2000). Molecular genetics: unmasking polyglutamine triggers in neurodegenerative disease.. Nat Rev Neurosci.

[2] Sapp E, Schwarz C, Chase K, Bhide PG, Young AB (1997). Huntingtin localization in brains of normal and Huntington's disease patients.. Ann Neurol.

[3] DiFiglia M, Sapp E, Chase KO, Davies SW, Bates GP (1997). Aggregation of huntingtin in neuronal intranuclear inclusions and dystrophic neurites in brain.. Science.

[4] Becher MW, Kotzuk JA, Sharp AH, Davies SW, Bates GP (1998). Intranuclear neuronal inclusions in Huntington's disease and dentatorubral and pallidoluysian atrophy: correlation between the density of inclusions and IT15 CAG triplet repeat length.. Neurobiol Dis.

[5] Davies SW, Turmaine M, Cozens BA, DiFiglia M, Sharp AH (1997). Formation of neuronal intranuclear inclusions underlies the neurological dysfunction in mice transgenic for the HD mutation.. Cell.

[6] Slow EJ, Graham RK, Osmand AP, Devon RS, Lu G (2005). Absence of behavioral abnormalities and neurodegeneration in vivo despite widespread neuronal huntingtin inclusions.. Proc Natl Acad Sci U S A.

[7] Ratovitski T, Gucek M, Jiang H, Chighladze E, Waldron E (2009). Mutant huntingtin N-terminal fragments of specific size mediate aggregation and toxicity in neuronal cells.. J Biol Chem.

[8] Graham RK, Deng Y, Carroll J, Vaid K, Cowan C (2010). Cleavage at the 586 amino acid caspase-6 site in mutant huntingtin influences caspase-6 activation in vivo.. J Neurosci.

[9] Butler DC, McLear JA, Messer A (2011). Engineered antibody therapies to counteract mutant huntingtin and related toxic intracellular proteins.. Progress in Neurobiology:.

[10] Messer A, Lynch SM, Butler DC (2009). Developing intrabodies for the therapeutic suppression of neurodegenerative pathology.. Expert Opin Biol Ther.

[11] Messer A, McLear J (2006). The therapeutic potential of intrabodies in neurologic disorders: focus on huntington and Parkinson diseases.. Biodrugs.

[12] Cardinale A, Biocca S (2008). The potential of intracellular antibodies for therapeutic targeting of protein-misfolding diseases.. Trends Mol Med.

[13] Perez-Martinez D, Tanaka T, Rabbitts TH (2010). Intracellular antibodies and cancer: new technologies offer therapeutic opportunities.. Bioessays.

[14] Atwal RS, Xia J, Pinchev D, Taylor J, Epand RM (2007). Huntingtin has a membrane association signal that can modulate huntingtin aggregation, nuclear entry and toxicity.. Hum Mol Genet.

[15] Cornett J, Cao F, Wang CE, Ross CA, Bates GP (2005). Polyglutamine expansion of huntingtin impairs its nuclear export.. Nat Genet.

[16] Omi K, Hachiya NS, Tanaka M, Tokunaga K, Kaneko K (2008). 14-3-3zeta is indispensable for aggregate formation of polyglutamine-expanded huntingtin protein.. Neurosci Lett.

[17] Rockabrand E, Slepko N, Pantalone A, Nukala VN, Kazantsev A (2007). The first 17 amino acids of Huntingtin modulate its sub-cellular localization, aggregation and effects on calcium homeostasis.. Hum Mol Genet.

[18] Kvam E, Nannenga BL, Wang MS, Jia Z, Sierks MR (2009). Conformational targeting of fibrillar polyglutamine proteins in live cells escalates aggregation and cytotoxicity.. PLoS One.

[19] Lecerf J-M, Shirley TL, Zhu Q, Kazantsev A, Amersdorfer P (2001). Human single-chain Fv intrabodies counteract in situ huntingtin aggregation in cellular models of Huntington's disease.. PNAS.

[20] Miller TW, Zhou C, Gines S, MacDonald ME, Mazarakis ND (2005). A human single-chain Fv intrabody preferentially targets amino-terminal huntingtin fragments in striatal models of Huntington's disease.. Neurobiol Dis.

[21] Murphy RC, Messer A (2004). A single-chain Fv intrabody provides functional protection against the effects of mutant protein in an organotypic slice culture model of Huntington's disease.. Brain Res Mol Brain Res.

[22] McLear JA, Lebrecht D, Messer A, Wolfgang WJ (2008). Combinational approach of intrabody with enhanced Hsp70 expression addresses multiple pathologies in a fly model of Huntington's disease.. FASEB J.

[23] Snyder-Keller A, McLear JA, Hathorn T Messer A Early or late-stage anti-N-terminal Huntingtin intrabody gene therapy reduces pathological features in B6.HDR6/1 mice.. J Neuropathol Exp Neurol.

[24] Ghoda L, van Daalen Wetters T, Macrae M, Ascherman D, Coffino P (1989). Prevention of rapid intracellular degradation of ODC by a carboxyl-terminal truncation.. Science.

[25] Li X, Zhao X, Fang Y, Jiang X, Duong T (1998). Generation of destabilized green fluorescent protein as a transcription reporter.. J Biol Chem.

[26] Leclerc GM, Boockfor FR, Faught WJ, Frawley LS (2000). Development of a destabilized firefly luciferase enzyme for measurement of gene expression.. Biotechniques.

[27] Sibler AP, Courtete J, Muller CD, Zeder-Lutz G, Weiss E (2005). Extended half-life upon binding of destabilized intrabodies allows specific detection of antigen in mammalian cells.. FEBS J.

[28] Ehrlich ME, Conti L, Toselli M, Taglietti L, Fiorillo E (2001). ST14A cells have properties of a medium-size spiny neuron.. Exp Neurol.

[29] Colby DW, Chu Y, Cassady JP, Duennwald M, Zazulak H (2004). Potent inhibition of huntingtin aggregation and cytotoxicity by a disulfide bond-free single-domain intracellular antibody.. Proc Natl Acad Sci U S A.

[30] Colby DW, Garg P, Holden T, Chao G, Webster JM (2004). Development of a human light chain variable domain (V(L)) intracellular antibody specific for the amino terminus of huntingtin via yeast surface display.. J Mol Biol.

[31] Weiss A, Klein C, Woodman B, Sathasivam K, Bibel M (2008). Sensitive biochemical aggregate detection reveals aggregation onset before symptom development in cellular and murine models of Huntington's disease.. J Neurochem.

[32] Kvam E, Sierks MR, Shoemaker CB, Messer A (2010). Physico-chemical determinants of soluble intrabody expression in mammalian cell cytoplasm.. Protein Eng Des Sel.

[33] Rechsteiner M, Rogers SW (1996). PEST sequences and regulation by proteolysis.. Trends Biochem Sci.

[34] Meng L, Mohan R, Kwok BH, Elofsson M, Sin N (1999). Epoxomicin, a potent and selective proteasome inhibitor, exhibits in vivo antiinflammatory activity.. Proc Natl Acad Sci U S A.

[35] Verhoef LG, Lindsten K, Masucci MG, Dantuma NP (2002). Aggregate formation inhibits proteasomal degradation of polyglutamine proteins.. Hum Mol Genet.

[36] Barkhordarian H, Emadi S, Schulz P, Sierks MR (2006). Isolating recombinant antibodies against specific protein morphologies using atomic force microscopy and phage display technologies.. Protein Eng Des Sel.

[37] Jana NR, Zemskov EA, Wang G, Nukina N (2001). Altered proteasomal function due to the expression of polyglutamine-expanded truncated N-terminal huntingtin induces apoptosis by caspase activation through mitochondrial cytochrome c release.. Hum Mol Genet.

[38] Southwell AL, Khoshnan A, Dunn DE, Bugg CW, Lo DC (2008). Intrabodies binding the proline-rich domains of mutant huntingtin increase its turnover and reduce neurotoxicity.. J Neurosci.

[39] Southwell AL, Ko J, Patterson PH (2009). Intrabody gene therapy ameliorates motor, cognitive, and neuropathological symptoms in multiple mouse models of Huntington's disease.. J Neurosci.

[40] Khoshnan A, Ko J, Patterson PH (2002). Effects of intracellular expression of anti-huntingtin antibodies of various specificities on mutant huntingtin aggregation and toxicity.. Proc Natl Acad Sci U S A.

[41] Greiner ER, Yang XW Huntington's disease: flipping a switch on huntingtin.. Nat Chem Biol.

[42] Wolfgang WJ, Miller TW, Webster JM, Huston JS, Thompson LM (2005). Suppression of Huntington's disease pathology in Drosophila by human single-chain Fv antibodies.. Proc Natl Acad Sci U S A.

[43] Zuccato C, Valenza M, Cattaneo E (2010). Molecular mechanisms and potential therapeutical targets in Huntington's disease.. Physiol Rev.

[44] Bett JS, Goellner GM, Woodman B, Pratt G, Rechsteiner M (2006). Proteasome impairment does not contribute to pathogenesis in R6/2 Huntington's disease mice: exclusion of proteasome activator REGgamma as a therapeutic target.. Hum Mol Genet.

[45] Bennett EJ, Shaler TA, Woodman B, Ryu KY, Zaitseva TS (2007). Global changes to the ubiquitin system in Huntington's disease.. Nature.

[46] Murakami Y, Matsufuji S, Hayashi SI, Tanahashi N, Tanaka K (1999). ATP-Dependent inactivation and sequestration of ornithine decarboxylase by the 26S proteasome are prerequisites for degradation.. Mol Cell Biol.

[47] Sarkar S, Rubinsztein DC (2008). Huntington's disease: degradation of mutant huntingtin by autophagy.. FEBS J.

[48] Auerbach W, Hurlbert MS, Hilditch-Maguire P, Wadghiri YZ, Wheeler VC (2001). The HD mutation causes progressive lethal neurological disease in mice expressing reduced levels of huntingtin.. Hum Mol Genet.

[49] Atwal RS, Xia J, Pinchev D, Taylor J, Epand RM (2007). Huntingtin has a membrane association signal that can modulate huntingtin aggregation, nuclear entry and toxicity.. Hum Mol Genet.

[50] Benn CL, Landles C, Li H, Strand AD, Woodman B (2005). Contribution of nuclear and extranuclear polyQ to neurological phenotypes in mouse models of Huntington's disease.. Hum Mol Genet.

[51] Landles C, Sathasivam K, Weiss A, Woodman B, Moffitt H (2010). Proteolysis of mutant huntingtin produces an exon 1 fragment that accumulates as an aggregated protein in neuronal nuclei in Huntington disease.. J Biol Chem.

[52] Schiefner A, Chatwell L, Korner J, Neumaier I, Colby DW A Disulfide-Free Single-Domain V(L) Intrabody with Blocking Activity towards Huntingtin Reveals a Novel Mode of Epitope Recognition.. J Mol Biol.

[53] Miller TW, Messer A (2005). Intrabody applications in neurological disorders: progress and future prospects.. Mol Ther.

[54] Martineau P, Jones P, Winter G (1998). Expression of an antibody fragment at high levels in the bacterial cytoplasm.. J Mol Biol.

[55] Sheets MD, Amersdorfer P, Finnern R, Sargent P, Lindquist E (1998). Efficient construction of a large nonimmune phage antibody library: the production of high-affinity human single-chain antibodies to protein antigens.. Proc Natl Acad Sci U S A.

[56] Tomlinson I, Holliger P (2000). Methods for generating multivalent and bispecific antibody fragments.. Methods Enzymol.

[57] Holt LJ, Bussow K, Walter G, Tomlinson IM (2000). By-passing selection: direct screening for antibody-antigen interactions using protein arrays.. Nucleic Acids Res.

[58] Melchionna T, Cattaneo A (2007). A protein silencing switch by ligand-induced proteasome-targeting intrabodies.. J Mol Biol.

[59] Steffan JS, Kazantsev A, Spasic-Boskovic O, Greenwald M, Zhu YZ (2000). The Huntington's disease protein interacts with p53 and CREB-binding protein and represses transcription.. Proc Natl Acad Sci U S A.

[60] Jach G, Pesch M, Richter K, Frings S, Uhrig JF (2006). An improved mRFP1 adds red to bimolecular fluorescence complementation.. Nat Methods.

